# Evaluation of a workplace suicide prevention program in the Australian manufacturing industry: protocol for a cluster-randomised trial of *MATES in manufacturing*

**DOI:** 10.1186/s12888-022-04464-3

**Published:** 2022-12-19

**Authors:** A. D. LaMontagne, L. L. Cox, C. Lockwood, A. Mackinnon, N. Hall, R. Brimelow, L. K.-D. Le, C. Mihalopoulos, T. King

**Affiliations:** 1 Institute for Health Transformation, Deakin University, Geelong, VIC Australia; 2 Richmond Fellowship, Toowoomba, QLD Australia; 3 MATES in Construction (National), Brisbane, Australia; 4 Centre for Mental Health, Melbourne School of Population & Global Health, University of Melbourne, Melbourne, Australia; 5 Black Dog Institute, University of New South Wales, Sydney, Australia; 6 Social Work & Communities, School of Social Sciences, Western Sydney University, Penrith, NSW Australia; 7 Department of Epidemiology & Preventive Medicine, Monash University, Melbourne, VIC Australia; 8 Centre for Health Equity, Melbourne School of Population & Global Health, University of Melbourne, Melbourne, VIC Australia

**Keywords:** Workplace, Suicide prevention, Manual workers, Mental health, Intervention, Cluster RCT, Evaluation

## Abstract

Males are at higher risk of death by suicide than females in Australia, and among men, blue-collar males are at higher risk compared to other working males. In response, MATES in Construction developed a workplace suicide prevention program for the construction sector in 2007 that has been widely implemented in Australia. In the current project, this program is being adapted and trialled in the manufacturing sector. The common aims of MATES programs are to improve suicide prevention literacy, help-seeking intentions, and helping behaviours. The program will be evaluated using a cluster randomised-controlled trial design with waitlist controls across up to 12 manufacturing worksites in Australia. We hypothesise that after 8 months of the MATES in Manufacturing program, there will be significantly greater improvements in help-seeking intentions (primary outcome) compared to waitlist controls. The project is led by Deakin University in collaboration with the University of Melbourne, and in partnership with MATES in Construction and a joint labour-management Steering Group.

**Trial registration:** The trial was registered retrospectively with the Australian New Zealand Clinical Trials Registry on 25 January 2022 (ACTRN12622000122752).

Protocol version: 2.0, November 2022.

**Supplementary Information:**

The online version contains supplementary material available at 10.1186/s12888-022-04464-3.

## Introduction

There are various work contexts where the workforce is predominantly male, particularly in manual, blue-collar work groups such as in construction, mining, energy and manufacturing. These are priority contexts for workplace suicide prevention for a number of reasons. Males are at higher risk of death by suicide than females: in Australia roughly three males die by suicide for every female; a pattern shared by many other developed countries [1]. Males are also less likely to seek help when distressed compared to females [2]. Hence, the gender composition of blue-collar manual occupational groups alone likely contributes to higher rates of suicide compared to other worker groups. Other evidence indicates that manual, blue-collar occupations and low educational attainment, including lower skill level within manual, blue-collar occupations (e.g., labourers versus skilled trades), are risk factors for suicide among men and young adults [3–8]. Further, particular working conditions endemic in male-dominated blue-collar industries such as construction, mining, energy and manufacturing are known to be harmful to mental health. These working conditions include long working hours, tight production timeframes, physically and psychologically demanding roles and duties, low job security, constrained autonomy, high injury rates, high prevalence of bullying, and limited mentorship and workplace support [9–12].

Protective factors for mental health and suicidality have also been documented amongst blue-collar workers. Good mentorship and patient, skilful supervision are protective factors for blue-collar workers, particularly apprentices [11, 13, 14]. Further, there is a growing evidence base that demonstrates the efficacy of the MATES in Construction workplace-based suicide prevention program for blue-collar and male-dominated industries. The MATES in Construction program was established by and for the Australian construction industry in 2007. It has also been adapted for the mining and energy sectors, and research evaluations in each sector have demonstrated the program’s positive impact on participants’ suicide prevention literacy, and willingness to seek and offer help [15–18]. However, the evidence to date has been limited to uncontrolled observational studies, with no experimental or randomised trial studies to date.

Based on a reasonable assumption that the MATES program will be relevant and likely effective in other blue-collar, male-dominated industries, the MATES program has been extended beyond the construction, mining and energy sectors, and is now being adapted for rollout in the manufacturing sector. The impetus for this suicide prevention program has come from within the manufacturing industry in the Australian state of New South Wales (NSW). Both employers and unions identified an unmet need for workers to receive support for suicidal distress, and jointly petitioned MATES in Construction to adapt its program for the manufacturing sector. MATES in Construction agreed to oversee the creation and implementation of the MATES in Manufacturing program, and to roll out the program in collaboration with a joint labour-management Steering Group in NSW.

Subsequent to the development of this initiative, funding was sought and obtained to evaluate the implementation and effectiveness of MATES in Manufacturing, hence leading to the development of this study protocol. This study will assess the implementation and effectiveness of the MATES program in the new context of manufacturing. Further, the proposed research employs a cluster-randomised controlled trial (cRCT) design, providing a much-needed complement to previously published observational evaluation evidence on MATES programs. If the results of this evaluation demonstrate the suitability and effectiveness of the MATES program in the NSW manufacturing sector, program reach could be further expanded to the broader Australian manufacturing sector and other blue-collar, male-dominated work contexts.

The following Research Questions (RQ) will be addressed using mixed methods for RQ 1–3 and quantitative methods for RQ 4–6:

1. Was the MATES in Manufacturing program implemented as designed?
2. What were the barriers and facilitators to participation in, and implementation of, MATES in Manufacturing?
3. What were participant and staff experiences of the program, including any noted outcomes arising (positive, negative, or other)?
4. Was suicide prevention literacy increased by the MATES in Manufacturing program relative to wait list controls? (process evaluation)
5. Did intentions to seek help increase in the MATES in Manufacturing program relative to wait list controls? (primary outcome)
6. Did help seeking behaviour increase, and levels of distress and suicidal ideation decrease, in the MATES in Manufacturing program relative to wait list controls? (secondary outcomes)

We also aim to evaluate the cost-effectiveness of the intervention if it is shown to have a significant positive effect on the primary outcome of help-seeking intentions.

## Methods

### Intervention description

**Fig. 1:**
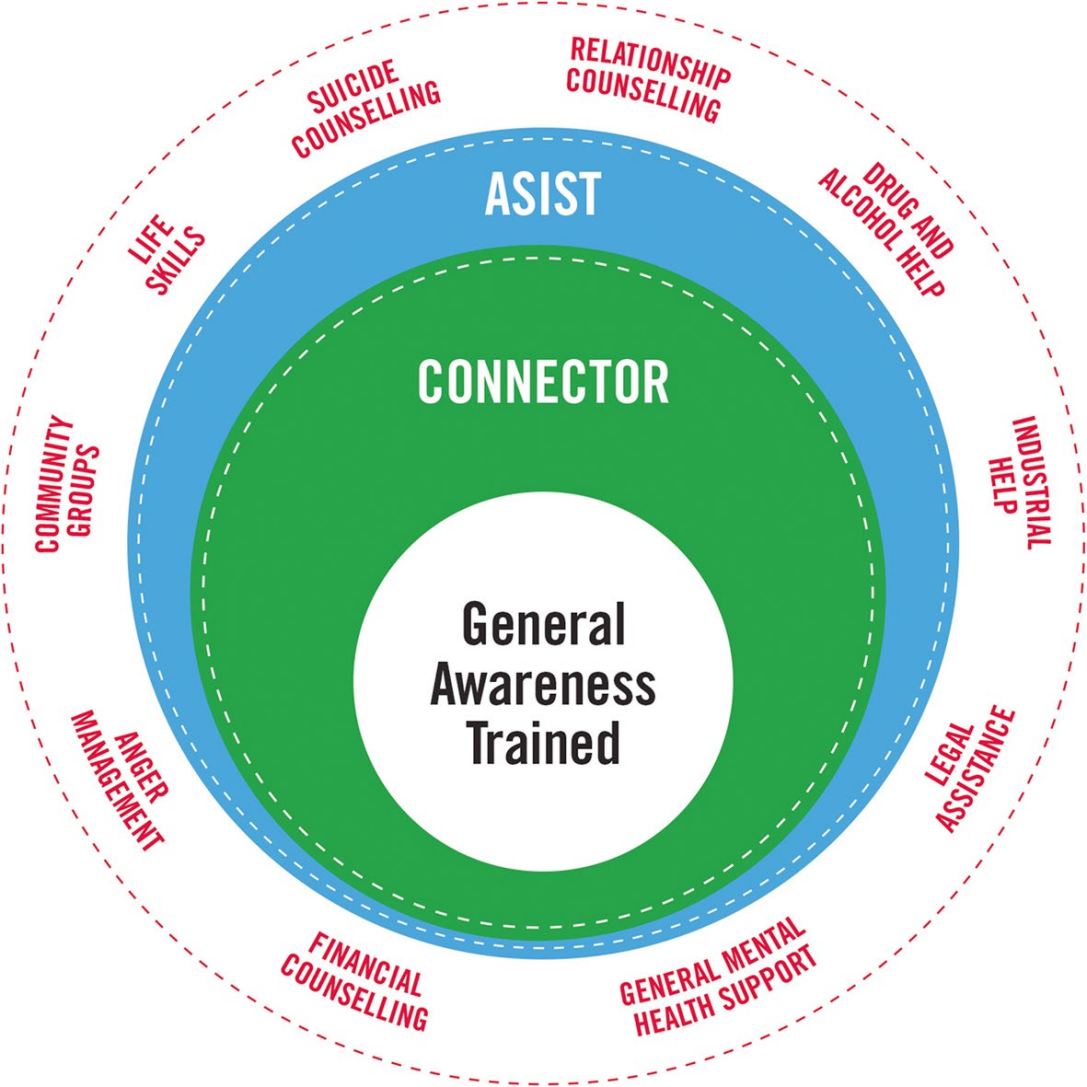
Schematic representation of MATES in Manufacturing workplace suicide prevention program: General Awareness trained workers (at centre) can access or be referred to Connectors and ASIST workers on site, and on-site supports are complemented and extended by MATES case managers who can further support and refer workers in need to a range of services (illustrative examples of services in outermost ring)

MATES in Manufacturing is an adaptation of the MATES in Construction workplace suicide prevention program (https://mates.org.au/). In brief, MATES is an industry-based community development approach to workplace mental health and suicide prevention. MATES does not provide clinical services but seeks to connect distressed workers to a range of clinical and non-clinical supports, as pictorially presented in Fig. 1.

Initially, a one-hour universal General Awareness Training (GAT) is provided for all workers on a site. Volunteers for half-day Living Works’ SafeTalk ‘Connector’ training are recruited during the GAT, and subsequently trained as the persons on site who can ‘connect’ workers in need to appropriate sources of help. Distressed workers can either approach a Connector him- or herself, or a Connector might approach someone based on observation, or concerns expressed by a workmate. ‘Help’ in this context is for mental health or suicidality, or for any issues that could contribute to distress, such as financial stress, alcohol and drug use, or relationship concerns. Volunteers are fundamental to the implementation of the MATES program, and the program aims to have an average of 1 in 20 workers on-site trained as Connectors. In addition to Connector training, MATES also delivers LivingWorks’ Applied Suicide Intervention Skills Training (ASIST): a 2 day session that equips participants with the skills to co-develop a safety plan with an individual at immediate risk of suicide. The program aims to have one to two ASIST-trained workers on each worksite.

Implementation of GAT on site is the starting point of the intervention. For those sites randomly assigned to intervention, the intervention will occur for 8 months during the study period, and then will continue beyond that timepoint at the discretion of the participating sites. Similarly, for sites randomly assigned to the wait-list control condition, the intervention will begin 8 months from the date of baseline assessment, and continue for at least 8 months thereafter. Participants in the trial will have indefinite access to MATES’ help line.

The MATES program is introduced to sites by Field Officers, who are employed directly by MATES. Field Officers provide GAT and ongoing support to MATES sites through regular site visits, establish and maintain relationships with workers on-site, and support site-specific Connectors in their role. In addition, MATES has a national toll-free helpline, through which workers and their immediate family members can be connected to case management support. Case managers are qualified to assist distressed workers with a plan to address their problems and concerns. This could include connecting workers with such services as their employer’s or union’s Employee Assistance Program (EAP), financial counselling, mental health services, drug and alcohol services, grief counselling, or family and relationship counselling.

The MATES in Manufacturing intervention will be delivered by the MATES in Construction NSW organisation.

### Evaluation study design

The MATES in Manufacturing intervention will be evaluated using a mixed methods implementation evaluation combined with a two-arm cluster-randomised trial design with wait-list controls for effectiveness evaluation. Implementation will be evaluated quantitatively by monitoring numbers and types of intervention activities and associated participation rates as well as suicide prevention literacy as a process evaluation measure. Qualitative implementation evaluation will entail characterising barriers and facilitators to implementation as well as participant experiences. Following SPIRIT Guidelines, the cRCT evaluation will assess help-seeking intentions as the primary outcomes, and help sought, suicidality, and psychological distress as secondary outcomes (see SPIRT Checklist, Supplementary File 1).

### Study setting & participants

The research project setting will be manufacturing worksites in the eastern Australia state of NSW that participate in the MATES in Manufacturing program.

#### Recruitment of study sites (clusters)

Manufacturing companies and sites will be recruited by MATES in Construction (National), led by Associate Investigators Lockwood, Cox, and Brimelow in collaboration with the joint labour-management Steering Group. The Steering Group includes representatives from MATES in Construction National, MATES in Construction NSW, the research team, interested manufacturing worksite employers, the Australian Industry Group (AIG) and unions representing workers at participating sites (Australian Manufacturing Union and Australian Workers Union).

#### Recruitment of participants within study sites (clusters)

All workers employed at each participating site will be eligible for the MATES program and all will be invited to participate in baseline and follow-up surveys (census sampling). We expect participants to be disproportionately male and blue-collar workers (estimated 75% males in Australian manufacturing sector). There will be two i*nclusion criteria: 1)* Employees or workers in the manufacturing industry who are working at participating sites of the MATES in Manufacturing program, including casual/temporary, contract, and continuing/permanent employees, and 2) Basic English proficiency for program activities (which will be conducted in English); however, surveys and some printed information on the program have also been translated into Vietnamese and Mandarin, and are available on an as-needed basis (some sites include a number of Vietnamese and Chinese workers). There is one e*xclusion criterion:* Individuals aged under 18 years.

### Sequence generation, randomisation and allocation concealment

Allocation of sites (clusters) to intervention arms will be conducted by the project statistician (AM) using minimisation implemented with the *rct_minim* procedure in Stata. Balance will be sought for a single factor – site size – with sites of less than 150 workers classified as ‘small’ and sites of 150 or more workers classed as ‘large.’ Due to resource limitations, participating sites will be randomly assigned to intervention or wait-list control in small batches of up to four sites (further detail below). Allocations will then be revealed to the research team, MATES field staff and sites, as blinding beyond the baseline assessments will not be feasible.

### Data collection (1): quantitative effectiveness evaluation

MATES will be the direct contact for recruitment, intervention, and data collection. All data will be collected by MATES Field Officers and research staff directly from workers, with no survey responses or data being shared with companies/employers. MATES Field Officers have access to and have been trained to adhere to a structured data collection protocol. All are experienced with data collection and have administered surveys as part of previous evaluation research projects.

Data collection will occur at each worksite, within work hours, at a date and time agreed with the company / site managers. Field Officers will deliver a brief preamble about the study purpose and aims. Workers will be informed that survey data collection and participation in MATES in Manufacturing as a program are separate processes: workers are welcome to participate in the program but are not compelled to participate in the surveys. There is no penalty for not completing a survey.

Workers will be given a plain language statement, consent form, and survey form to peruse, and the consent process will be explained verbally. Interested workers will be asked to sign the consent form to confirm their willingness to participate, and Field Officers will answer any questions arising. Participants will be invited to seal their consent form and survey in a provided envelope (with or without responses) and place it into a data collection box. This will help ensure that those who do not wish to participate in surveys will not be readily identifiable. See Supplementary File 2 for Plain Language Statements and Consent Forms.

At the end of the site visit, the data collection box will be removed from the site by MATES Field Staff. This box will then be transported, by courier, to the MATES Research Manager who is based in the Queensland office, where it will be entered into a secure electronic database. Survey responses will not be handled by or made available to employers.

Data for the economic evaluation will be collected as a supplementary survey at time 2 (8 months) for both intervention and wait-list control sites. Following administration of the paper ‘core’ survey, participants will be invited to scan a QR code and complete a brief health service use questionnaire.

#### Data collection safety measures

Because respondents will be asked sensitive questions (e.g., about suicide), we prepared a safety protocol for data collection and intervention activities. We have three mechanisms in place for following up with participants who indicate (either verbally or through physical non-verbal cues) that they are upset or in distress at the time of data collection or during program activities, and for following up with participants who indicate through their survey responses that they have recently experienced psychological distress:

1. Field Officers will observe participants throughout survey administration. Anyone who appears to be uncomfortable or upset will be offered immediate support. All Field Officers have completed Applied Suicide Intervention Skills Training, and all are experienced with supporting individuals in distress;
2. At the end of their survey (tick box question on survey) or by direct in-person request, participants will be able to indicate that they would like a follow-up support call from MATES. This is a mechanism that all MATES programs use during training and in survey administration for other program evaluation projects (participants can self-nominate whether they would like a follow up call after training sessions). All requests for follow up calls will be addressed immediately;
3. As soon as surveys are returned, the Research Manager will review responses for any indications that a participant is in distress, or has recently been in distress (e. g., ‘rather likely’ or ‘very likely’ responses to the question “How likely is it that you will attempt suicide someday?”). This too will trigger immediate follow up via phone, and referral to MATES case management staff.

#### Data collection timing

**Fig. 2:**
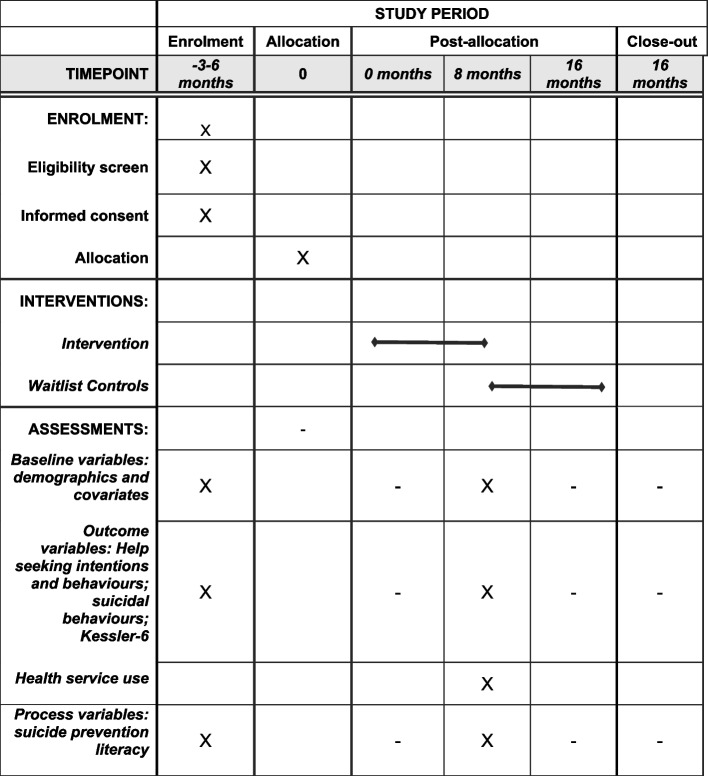
MATES in Manufacturing cluster RCT: schedule of enrolment, interventions, and assessments

Intervention sites will participate in baseline surveys then 8 months of the MATES in Manufacturing program from the first GAT on site, with follow-up surveys administered 8 months after intervention initiation. Wait-list control sites will participate in baseline surveys (month 0) then follow-up surveys 8 months later. At wait-list control sites, the MATES program will commence following the 8 month surveys, then continue for at least 8 months. See Fig. 2: schedule of enrolment, interventions, and assessments.

### Data collection (2): qualitative implementation evaluation

Volunteers for Connector and ASIST roles at participating sites will be recruited through posters with information provided for contacting the research team (phone numbers and QR codes). Program participants (including those recruited to be Connectors and ASIST workers) and Field Officers will be interviewed in separate focus groups or individual interviews, as is feasible for scheduling. We will aim to conduct focus groups in person, but depending on the state of COVID-19 restrictions at the time, we may conduct individual or focus group interviews by Zoom.

Informed consent will be provided on recruitment and scheduling of interviews. The same safety protocol described for the surveys will be applied in the context of focus group interviews.

### Measures (1): effectiveness evaluation

#### Help-seeking sources & intentions (primary outcome)

Help-seeking intentions will be measured using the General Help-seeking Behaviour Questionnaire (GHSQ). GSHQ questions will be modified to present 12 different sources of help seeking (including a MATES worker or Connector and an open-ended option for ‘other’), and participants will be asked to rank their help-seeking intentions prefaced by “if you were feeling overwhelmed and unable to cope.” This phrasing was derived from a qualitative study on how blue-collar male workers conceptualise emotional and suicidal distress, with the results indicating that blue collar male workers are inclined to emphasise a loss of agency [19]. Intentions will be ranked from *extremely unlikely* to *extremely likely on a 7-point scale* [20]. A single summary measure across all the 12 options will be computed by summing the responses (reverse scoring the ‘no one’ option).

#### Help sought (secondary outcome)

Among those who report having felt overwhelmed or unable to cope at any point in the preceding 6 months (yes/no), forms of help sought during the preceding 8 months will be measured using the General Help-seeking Behaviour Questionnaire (GHSQ). GSHQ questions will list the same 12 sources of help as detailed in the primary outcome of help-seeking intentions, except in this case asking whether help has been sought, and how frequently (never/rarely/sometimes/often/always). A single summary measure across all the 12 options will be computed by summing the responses (reverse coding the ‘no one’ option).

#### Suicidal behaviour (secondary outcome)

Suicidal behaviour will be measured using 2-items from the 4-item version of the Suicidal Behaviour Questionnaire-Revised (SBQ-R) [21]. The first item assesses the frequency of suicidal ideation in the past 6 months, and responses will be dichotomised (never/ever). The second item measures the likelihood of suicidal behaviour in the future (no chance/unlikely/likely).

#### Psychological distress (secondary outcome)

Psychological distress will be measured using the Kessler-6 instrument (K6) [22]. Participants will be asked to indicate the response that best describes their feelings in the past 4 weeks. Responses will be made on a 5-point Likert scale ranging from 0 (*none of the time*) to 4 (*all of the time*). The six items will be summed to give a score ranging from 0 to 24.

#### Health service use (economic evaluation)

Health service use will be assessed using a modified version of the self-report measure of the Resource Use Questionnaire [23] developed for use in this study. Five questions will assess participants’ use of a range of health services for mental health difficulties over the preceding 6 months, including primary health services (e.g. consultations with a general practitioner, psychologist) and inpatient admissions (e.g. hospital, community care unit). Use of psychotropic medications (e.g. sleeping tablets, antidepressants) is also assessed, as is the effect of mental health difficulties on functioning at work and absence from work.

#### Covariates

Name & mobile telephone contact number (for within-person linking of baseline and final assessments), company/site name (for cluster identification), age, gender, occupation, Aboriginal and Torres Strait Islander status, Australian-born versus born outside Australia, history of previous training in MATES programs (Construction, Energy, or Mining).

### Measures (2): implementation evaluation

We will monitor and record the number of program activities and participation levels in program activities (e.g., percent of workers on site attending GAT; number and percent of GAT trainees recruited and successfully trained as Connectors). In addition, we will measure suicide prevention literacy as a process/implementation measure using the literacy of suicide scale short form (LOSS). Each of the items on the LOSS is responded to on a 3-point scale (“True”, “False”, or “I don’t know”), with correct responses allocated a score of 1 and incorrect or “I don’t know” responses assigned a score of 0 (e.g., sample item: “People who have thoughts about suicide should not tell others about it”). Total scale scores are calculated by summing item scores, yielding a total literacy score (per cent correct). The LOSS has been validated by using Item Response Theory to identify items that had the strongest discrimination of the underlying literacy construct [24].

### Analysis (1): quantitative effectiveness evaluation

The primary analysis will be undertaken on an intention-to-treat basis: including all participants as randomised, regardless of treatment received or withdrawal from the study. Mixed-model Repeated Measures (MMRM) analyses will be used because of the ability of this approach to include participants with missing data. Cluster effects will be modelled using a random site effect. An unstructured residual variance-covariance matrix will accommodate within-participant dependency. The statistical significance of the primary outcome will be evaluated in a planned comparison of change from baseline to 8 months post-initiation of intervention in the active arm compared to 8-month change in the waitlist group. Tests of significance will use appropriate degrees of freedom adjustment where necessary (i.e., the Kenward-Roger method based on the observed information matrix). Where necessary, transformation of the outcome variable will be undertaken to ensure distributional assumptions of the model are met.

For binary outcomes (prevalence of suicidal ideation) and other non-continuous outcomes (severity of suicidal thoughts and behaviours), comparable generalized mixed models will be used with population average effects of intervention being compared.

#### Power and sample size

We based our power and sample size calculations on the primary outcomes: intentions to seek help from formal sources. From a previous MATES trial in construction workers (“MATES Mobile,” ACTRN12619000625178) [25], we obtained clustered data with which to estimate intra-class correlation (ICC) and the corresponding design effect. Our measure of intention to seek help for emotional problems from formal sources yielded an ICC estimate of 0.0112. To be conservative, we used an ICC of 0.02. For our anticipated effect size, or Minimally Important Difference (Cohen’s d, standardised difference in change between intervention and control groups in a two-arm trial), we have used 0.30; this effect size is modest, but is in the range of what has been achieved in previous workplace suicide prevention trials. Other inputs into power calculations included: alpha = 0.05 and beta = 0.80. Because we did not know in advance what our cluster sizes would be, a set of calculations was conducted assuming a range of anticipated cluster sizes (30, 50 and 100 workers per cluster/site). At the lower cluster size of 30, we would need 19 sites, at the middle cluster size of 50 we would need 14 sites, and finally if our cluster sizes average 100 or more, we would need only 10 sites. Hence the target sample size in this protocol is estimated at 1000, based on recruiting 10 sites of 100 workers each, acknowledging that the final sample size may vary based on worksite size.

In addition, we will conduct exploratory analyses to investigate whether intervention-associated changes differ by gender or by manual blue-collar versus white-collar.

### Analysis (2): implementation evaluation

Descriptive analyses will be reported on frequencies of program intervention activities relative to plans, and participation levels in program activities.

Interview transcripts will be analysed using descriptive thematic analysis [26] to answer the following Research Questions:

1. Was the MATES in Manufacturing program implemented as designed?
2. What were the barriers and facilitators to participation in, and implementation of, MATES in Manufacturing?
3. What were participant and staff experiences of the program, including any noted outcomes arising (positive, negative, or other)?

The suicide prevention literacy process measures collected by survey will be analysed as above for the quantitative effectiveness evaluation, Research Question 4:

4) Was suicide prevention literacy increased by the MATES in Manufacturing program relative to wait list controls? (process evaluation)

### Analysis (3): economic evaluation

If the trial achieves significant positive improvements in the primary outcome of help-seeking intentions, a trial-based economic evaluation will be conducted based on the help-seeking intention scores, the intervention costs, and mental health-related services costs. Intervention costs include the intervention development costs (e.g. planning, development, and materials). Mental health services cost will be calculated by applying standard Australian unit costs (i.e. Independent Hospital Pricing Authority [27], Medicare Benefit Scheme fees [28]) to the resource use units collected through the HSUQ. Mean values of costs and help-seeking intention scores will be reported for both groups and assessed by generalised linear models. An incremental cost-effectiveness ratio (ICER) will be calculated as the difference in average cost between the groups, divided by the difference in help-seeking intention scores. Nonparametric bootstrapping will be used to obtain confidence intervals for incremental cost-effectiveness ratios. A modelled economic evaluation will also be undertaken using the results of this trial and relevant epidemiological literature to extrapolate long-term costs and consequences associated with help-seeking intentions.

### Ethics approvals and monitoring

Ethics approval for the cRCT was granted on 3 September 2021 by the Deakin University Human Research Ethics Committee (protocol number 2021–276), with approval for the qualitative implementation evaluation on 30 September 2022 from the Western Sydney University Human Research Ethics Committee (protocol number H14506). See Supplementary File 3 for Ethics Approvals. The research will be subject to compliance monitoring and auditing by these two university Ethics Committees. In addition, the lead investigator and MATES staff report biweekly on trial progress to the meeting of the joint labour-management Steering Group. The Steering Group was initiated in 2020, and the trial lead investigator has been participating since early 2021, in the lead up to site recruitment. Progress of the trial, implementation of intervention activities, and any concerns arising from participating company and union representatives and MATES staff are discussed. MATES reports on the uptake and usage of the MATES 1–800 and case management services (aggregate usage only, no company identifiers), and participating companies and unions report on their experiences of the trial and MATES program to date. We do not have ‘trial stopping’ guidelines in place, but do have a Withdrawal of Consent process for participating companies (see Supplementary File 1). These monitoring functions operate independently of the funder of the research. There is no trial sponsor and there is no formal Data Monitoring Committee; these were deemed unnecessary because this a community-based and not a clinical trial.

### Trial status (as of submission)

Site recruitment commenced in December of 2021, but has been hampered by the COVID-19 pandemic and the associated restrictions and impacts on businesses. As sites were recruited, they were grouped and randomly assigned as soon as feasible, such that data collection and intervention implementation could be spread out over time and managed with the small implementation and research teams available. Two groups of four sites have been recruited and baseline data collection completed before random assignment by the procedure detailed above (running total = 5 sites assigned to intervention and 3 sites to wait-list control). Intervention activities have been initiated at most sites assigned to the intervention condition. A third and final group of sites awaits completion of baseline surveys and random assignment. The 8 sites recruited to date vary widely in size, ranging from 25 up to 440 workers, corresponding to an estimated study population of 1573 workers in total. Survey participation to date has been relatively high, with an overall average of 74% and a range of 60–92% over the 8 sites.

Though few in number, distressed workers have been identified and followed up while piloting the survey, and at both intervention and wait-list control sites during the conduct of baseline surveys; the frequency and nature of these events will be captured from Field Officer notes. While strictly speaking, this would constitute ‘contamination’ of wait-list control sites, this is a limitation that is ethically imperative and is not likely to affect the overall results of the trial.

### Dissemination

For study participants, the final results of the trial RCT will be presented to the joint labour-management Steering Group, and we will provide a plain English lay summary for distribution to program participants by management and union steering group members. For the research, policy, and practice communities, we will publish peer-reviewed journal articles and deliver presentations at national and international research conferences. We will also deliver other presentations on findings to mental health and suicide prevention practitioners and policymakers.

## Discussion

This project represents a strong partnership bringing together practitioners at the coalface of suicide prevention (MATES), industry partners (participating sites, unions, and Steering Group), and suicide prevention researchers. Further, the trial addresses an urgent need for experimental evidence on the effectiveness of workplace suicide prevention programs, both for MATES in particular as well as for workplace suicide prevention in general [29]. The qualitative implementation evaluation aims to provide narrative insights into how the intervention gets implemented: what it “looks like” in practice [30]. The qualitative implementation evaluation will be complemented by quantitative data collection focussed on the extent to which program components were implemented as planned as well as the extent of participation in intervention activities. The combination of qualitative and quantitative methods will enable the exploration of context-specific factors that could modify intervention implementation as well as effectiveness [31].

While the cRCT design provides strong internal validity, our study may be limited with respect to external validity, or generalisability. Worksites that have engaged with the labour-management steering committee and are willing to accept the terms of an experimental study may be a select group that is not representative of all manufacturing worksites. Within sites, however, we expect that the potential for selection bias is limited, given the relatively high participation rates in the baseline surveys (average = 74%), which we will hope to retain at follow-up.

In addition to contributing to the international literature on workplace suicide prevention, the trial will generate evidence-based policy and practice insights for the partners involved. The MATES intervention is being implemented under real world conditions by an established workplace suicide prevention charity, driven by a need identified by industry partners; hence the potential for translation of this research to policy and practice is high. The project partners are well positioned to adapt and disseminate findings to a wide range of workplace mental health and suicide prevention stakeholders. The MATES workplace suicide prevention strategy under evaluation could also be adapted to other settings nationally and internationally if the findings are positive, and at a minimum will provide implementation and effectiveness insights for future research, policy and practice.

## Supplementary Information

**Additional file 1.** SPIRIT 2013 Checklist: Recommended items to address in a clinical trial protocol and related documents*

**Additional file 2.**

**Additional file 3.**

## Data Availability

The datasets used in the current study are available from the corresponding author on reasonable request. Anonymized, participant-level data may be made available to external researchers on the basis of a scientifically sound proposal, subject to ethics approval. The study protocol will be available at the Australian New Zealand Clinical Trials Registry (ANZCTR). Any amendments to the protocol will be provided to the ANZCTR and will also be communicated to all trial researchers.

## Abbreviation

cRCT: Cluster-randomised, controlled trial
