# Correction: Evaluation of a workplace suicide prevention program in the Australian manufacturing industry: protocol for a cluster-randomised trial of *MATES in manufacturing*

**DOI:** 10.1186/s12888-023-04590-6

**Published:** 2023-02-08

**Authors:** A. D. LaMontagne, L. L. Cox, C. Lockwood, A. Mackinnon, N. Hall, R. Brimelow, L. K.-D. Le, C. Mihalopoulos, T. King

**Affiliations:** 1 Institute for Health Transformation, Deakin University, Geelong, VIC Australia; 2 Richmond Fellowship, Toowoomba, QLD Australia; 3 MATES in Construction (National), Brisbane, Australia; 4 Centre for Mental Health, Melbourne School of Population & Global Health, University of Melbourne, Melbourne, Australia; 5 Black Dog Institute, University of New South Wales, Sydney, Australia; 6 Social Work & Communities, School of Social Sciences, Western Sydney University, Penrith, NSW Australia; 7 Department of Epidemiology & Preventive Medicine, Monash University, Melbourne, VIC Australia; 8 Centre for Health Equity, Melbourne School of Population & Global Health, University of Melbourne, Melbourne, VIC Australia

**Correction: BMC Psychiatry 22, 799 (2022)**

**https://doi.org/10.1186/s12888-022-04464-3**

Following publication of the original article [1], the authors identified that R. Brimelow and C. Lockwood were incorrectly assigned to affiliation 2. The authors are assigned to affiliation 3.

The original article [1] has been corrected.

## References

[1] LaMontagne (2022). Evaluation of a workplace suicide prevention program in the Australian manufacturing industry: protocol for a cluster-randomised trial of MATES in manufacturing. BMC Psychiatry.

