# Supplementary material for: Evaluation of a workplace suicide prevention program in the Australian manufacturing industry: protocol for a cluster-randomised trial of MATES in manufacturing
Source: BMC Psychiatry. 2022 Dec 19;22:799. doi: 10.1186/s12888-022-04464-3 (PMC9761021; doi:10.1186/s12888-022-04464-3)
Supplement: Supplementary file 2 — Additional file 2. [file 12888_2022_4464_MOESM2_ESM.pdf]

Plain Language Statement

Project: MATES in Manufacturing

Professor Anthony LaMontagne (Responsible Researcher)
Tel: +61 3 9244 6802   Email: tony.lamontagne@deakin.edu.au

Introduction
Thank you for your interest in participating in this research project. The following few pages will provide you with further information about the project, so that you can decide if you would like to take part.

Please take the time to read this information carefully. You may ask questions about anything you don't understand or want to know more about.

Your participation is voluntary. If you don't wish to take part, you don't have to. If you begin participating, you can also stop at any time.

What is this research project all about?
The project seeks to evaluate whether the MATES in Manufacturing program improves suicide prevention literacy, help-seeking, and help-offering behaviours among workers in the manufacturing sector. There will also be a supplementary survey on health service use to evaluate the economic impacts of the MATES program. The research team includes Professor Anthony LaMontagne (Deakin University), Dr Tania King (University of Melbourne), Dr Neil Hall (University of Western Sydney), and Ms. Rachel Brimelow (MATES in Construction) and Chris Lockwood (MATES in Construction).

Participation procedure
Should you agree to participate, you will complete surveys at up to three points in time. The "core" survey will include questions to assess knowledge and attitudes about suicide prevention, suicide ideation, self-harm, general help-seeking behaviours, and social support structures. These first "core" survey is to be completed on paper (hard copy) when you first begin participating. The later surveys will include a repeat of the "core" survey on paper, and will additionally invite you to complete questions on-line about your use of health services, including consultations with General Practitioners (GPs), psychologists, and other healthcare providers.. All surveys will be distributed, and hard copies collected at your workplace by a member of the research team. On-line surveys go directly to the research team.

Survey responses will not be handled by or made available to employers. The "core" survey is our first priority for the research, because it will be used to see if the program works. The on-line supplemental survey about health service use would additionally allow us to evaluate the economic impacts of the MATES program. Both surveys are voluntary. You are welcome to participate in both, one and not the other, or neither of the surveys.

Consent
You will be provided with a consent form prior to participating in this research project. In signing and returning the consent form to a member of the research team you will be acknowledging that you voluntarily agree to participate, and that you give consent for your data to be used for the research purposes described above.

What are the possible benefits?
By participating in this research, you will be contributing to our understanding of suicide literacy, help-seeking, and help-offering behaviours. This knowledge can be used to inform future development of workplace suicide prevention programs specific to the manufacturing sector. Individuals who show indications of acute distress on baseline surveys will be offered help by MATES staff (which you may choose to accept or not).

What are the possible risks?
Your participation in this research project requires your commitment of time, which may pose inconvenience to you. To minimise this inconvenience, we are doing survey on work time and expect that each survey should only take about 10-15 minutes to complete.

Thinking about suicide and self-harm may cause feelings of distress. If you do feel distress, you can contact the MATES 24/7 National Helpline (1300 642 111) or Lifeline (13 11 14). You will be provided with support and connected to outside sources of counselling.

We recognise that participating in this research requires you to gather in-person with your co-workers. To help keep participation COVID safe, the research team will keep up to date with the Chief Health Officer's public health directions, and we will make any necessary adjustments to the project as circumstances change. We will only conduct surveys in locations at your worksite where all participants are able to observe physical distancing requirements. We will ensure hand sanitisers are available for use, and we will have disposable masks on hand, should you like one.

What will happen to information about me?
Your information will be kept confidential. During the project, only the Project Manager will have access to your personal information. Any data transferred between researchers across collaborating universities will only be done using password-protected cloud storage and with all identifying information removed. Project data will be kept on a password-protected computer in a secure location at Deakin University. We will keep this data for 5 years after the last publication arising from the project, at which point it will be permanently deleted. This data is critical to ongoing evaluation of suicide prevention interventions, and efforts to save lives in the manufacturing industry rest on having good data. Therefore, the data may be used again by the research team for future evaluation efforts about suicide prevention among people working in manufacturing.

Do I have to take part?
No. Participation is completely voluntary. You have the right to choose not to participate and are able to withdraw at any time without penalty. Once data has been collected, it will be analysed and the results will be included in publications and presentations. However, if you choose to withdraw from the project at any point, any unprocessed data that you have provided will not be included in publications and presentations.

Will I hear about the results of this project?
On completion of this project, we will provide a plan English summary of outcomes to all interested participants both through the participating companies and unions. We will also use data gathered during this project to publish articles in academic journals, present at conferences, and to advocate for improvements in suicide prevention policy and practice.

Who is funding this project?
This project is funded by a *National Health and Medical Research Council Million Minds, Mission Suicide Prevention 2019* grant.

Where can I get further information?
If you have any questions, or would like more information, please contact responsible researcher, Professor Tony LaMontagne (03 9244 6802; tony.lamontagne@deakin.edu.au).

Who can I contact if I have any concerns about the project?
This research project has been approved by the Human Research Ethics Committee of Deakin University (ID #2021-276). If you have any concerns or complaints about the conduct of this research project, which you do not wish to discuss with the research team, you should contact the If you have any complaints about any aspect of the project, the way it is being conducted or any questions about your rights as a research participant, then you may contact: The Human Research Ethics Office, Deakin University, 221 Burwood Highway, Burwood Victoria 3125, Telephone: 9251 7129, research-ethics@deakin.edu.au. Please quote project number [2021-**276]**. All complaints will be treated confidentially. In any correspondence, please provide the name of the research team or the name or ethics approval number of the research project.

Participant Consent Form

MATES in Manufacturing: A Workplace Suicide Prevention Cluster RCT

Responsible Researcher: Professor Anthony LaMontagne (Deakin University)

Additional Researchers: Dr Tania King (University of Melbourne), Dr Neil Hall (Western Sydney University), Ms Rachel Brimelow (MATES in Construction) and Chris Lockwood (MATES in Construction)

Name of Participant: ____

Mobile Number: ____

1. I consent to participate in this project, the details of which have been explained to me, and I have been provided with a written plain language statement to keep.
2. I understand that the purpose of this research is about suicide literacy, help-seeking, and help-offering behaviours among people employed in the manufacturing sector.
3. I understand that my participation in this project is for research purposes only.
4. I acknowledge that the possible effects of participating in this research project have been explained to my satisfaction.
5. In this project I will be required to complete an initial paper-based survey which will be distributed by a member of the research team at my workplace. I will also complete a follow-up survey at 8 months.
6. I understand that my participation is voluntary and that I am free to withdraw from this project at any time without explanation or prejudice, and to withdraw any unprocessed data that I have provided.
7. I understand that the data from this research will be stored at Deakin University and will be retained for at least 5 years after the last publication is released from this project.
8. I have been informed that this research will be conducted in line with COVID safe requirements, and that COVID safe measures will be adjusted in line with the Chief Health Officer's public health directions, as these are issued.
9. I have been informed that the confidentiality of the information I provide will be safeguarded subject to any legal requirements; my data will be password protected and accessible only by the named researchers. Any data transferred between researchers across different universities will only be done so using cloud storage and with all identifying information removed.
10. I understand that because my mobile phone number will be used to link my survey responses, it is not possible to guarantee my anonymity. However, no information that could lead to my identification will be disclosed in any reports on the project, or to any other party.

11. I understand that after I sign and return this consent form, it will be retained by the researcher.

12. I understand that this study has received Deakin University ethics approval (reference number: 2021-276)

Participant Signature: **Date:**

Finally, in the future there may be opportunities for the research team to conduct follow-up research that draws on the MATES in Manufacturing research evaluation data. Please check the below box if you consent for your data to be used in later follow-up research of a similar nature:

☐ I give consent for my data to be used in follow-up research

MATES in Manufacturing: a workplace suicide prevention intervention

Supplementary survey – Evaluating the economic impacts of MATES in Manufacturing.

This supplementary survey will enable us to evaluate the economic impacts of the MATES in Manufacturing suicide prevention program. The paper survey contained within this survey pack is the 'core' survey for this research study. This additional online survey asks questions about your use of health services, including consultations with General Practitioners (GPs), psychologists, and other healthcare providers. As for the hard copy 'core' survey, your responses to the on-line supplementary survey go directly to the research team.

Survey responses will not be handled by, or made available to, employers. Both surveys are voluntary. You are welcome to participate in both, one and not the other, or neither of the surveys.

To access the full Plain Language Statement regarding the project aims, procedures, benefits and risks please refer to the MATES webpage:

Mates.org.au/manufacturingtrial

If you have any questions, or would like more information, please contact responsible researcher, Professor Tony LaMontagne (03 9244 6802; tony.lamontagne@deakin.edu.au).

If you have any concerns or complaints about the conduct of this research project, which you do not wish to discuss with the research team, you may contact: The Human Research Ethics Office, Deakin University, Telephone: 9251 7129, research-ethics@deakin.edu.au. Please quote project number [2021-**276**].

Your QR Code

Scan this QR code to participate.

Mates in Manufacturing Suicide Prevention Project

Participant Information Sheet

This research is about understanding the experiences of participants in the MATES in Manufacturing program. Specifically, we want to understand how the program impacts people's awareness of mental health issues, knowing where to go for help, and how best to offer help to other workers. Western Sydney University Human Research Ethics Committee has approved the research (number **H14506**).

What will I be asked to do?
You are invited to participate in an interview, either face-to-face, by phone or on Zoom, to understand your experience of the MATES in Manufacturing program.

How much of my time will I need to give?
It may take 30 to 45 minutes to complete the interview.

What benefits will I, and/or the broader community, receive for participating?
By participating in this research, you will be contributing to our understanding of how to prevent suicide among manufacturing workers. This knowledge can be used to inform future development of workplace suicide prevention programs specific to the manufacturing sector.

Will the study involve any risk or discomfort for me? If so, what will be done to rectify it?
We do not anticipate that participating in this project will cause discomfort. However, thinking about suicide and self-harm may cause feelings of distress. If you do feel distress, you can contact the worksite Connectors, MATES Case Managers or call the MATES 24/7 National Helpline (1300 642 111) or Lifeline (13 11 14). You will be provided with support and connected to outside sources of counselling if needed.

How do we intend to publish the results?
On completion of this project, we will provide a plain English summary of outcomes to all interested participants both through the participating companies and unions. We will also use data gathered during this project to publish articles in academic journals, present at conferences, and to advocate for improvements in suicide prevention policy and practice.

When will the data and information that I have provided be disposed of?
Only the researchers will have access to the information you provide. Data will be stored electronically under password security on the Western Sydney University network for up to 8 years before being permanently deleted.

Can I withdraw from the study?
Yes. Participation is entirely voluntary and you are not obliged to be involved. If you do participate you can withdraw at any time without giving reason. If you choose to withdraw, any information that you have supplied will be removed from the study.

What if I require further information?
If you would like to discuss the research further before deciding whether to participate, please contact Dr Neil Hall on 0417 278 645 or email: n.hall@westernsydney.edu.au

What if I have a complaint?
If you have any complaints or reservations about the ethical conduct of this research, you may contact the Ethics Committee through Research Engagement, Development and Innovation (REDI) on Tel +61 2 4736 0229 or email humanethics@westernsydney.edu.au. Any issues you raise will be treated in confidence and investigated fully, and you will be informed of the outcome.

If you agree to participate in this study, you will be asked to sign the Participant Consent Form. The information sheet is for you to keep and the consent form is retained by the researcher/s.

WESTERN SYDNEY
UNIVERSITY

Consent Form

Mates in Manufacturing Suicide Prevention Project: Measuring the impact of on-site peer-to-peer mental health training to improve help-seeking and help-offering behaviour

This study has been approved by the Human Research Ethics Committee at Western Sydney University. The ethics reference number is: H*14506*

I hereby consent to participate in the above named research project.

I acknowledge that:

- I have read the participant information sheet (or where appropriate, have had it read to me) and have been given the opportunity to discuss the information and my involvement in the project with the researcher/s

- The procedures required for the project and the time involved have been explained to me, and any questions I have about the project have been answered to my satisfaction.

I consent to:

☐ *Participating in an interview*

☐ *Having my information audio recorded*

I consent for my data and information provided to be used in this project and other related projects for an extended period of time.

I understand that my involvement is confidential, and that the information gained during the study may be published and stored for other research use but no information about me will be used in any way that reveals my identity.

I understand that I can withdraw from the study at any time without affecting my relationship with the researcher/s, and any organisations involved, now or in the future.

Signed:

Name:

Date:

Return Address: n.hall@westernsydney.edu.au or in person

What if I have a complaint?

If you have any complaints or reservations about the ethical conduct of this research, you may contact the Ethics Committee through Research Engagement, Development and Innovation (REDI) on Tel +61 2 4736 0229 or email humanethics@westernsydney.edu.au.

Any issues you raise will be treated in confidence and investigated fully, and you will be informed of the outcome.

Plain Language Statement for Companies

Project: MATES in Manufacturing: A Workplace Suicide Prevention Cluster RCT

Professor Anthony LaMontagne (Responsible Researcher)
Tel: +61 3 9244 6802   Email: tony.lamontagne@deakin.edu.au

Introduction
Thank you for your interest in participating in this research project. The following few pages will provide you with further information about the project, so that you can decide if you would like for your company to participate.

If you have any questions about the research or if you would like to discuss any aspects of the research process, please contact Professor Tony LaMontagne via the details above.

The participation of your company in this research project is connected to your participation in the MATES in Manufacturing pilot program that is being trialled by MATES in Construction: your company's participation in the pilot is contingent on your participation in this research evaluation.

Your participation in the pilot program and associated research evaluation is voluntary. If you wish to withdraw from participating in the MATES in Manufacturing pilot and research evaluation, you can do so at any time.

What is this research project all about?
The project seeks to evaluate whether the MATES in Manufacturing program improves suicide prevention literacy, help-seeking, and help-offering behaviours among workers in the manufacturing sector. The research team includes Professor Anthony LaMontagne (Deakin University), Dr Tania King (Univeristy of Melbourne), Dr Neil Hall (Univiestiy of Western Sydney), Dr Laura Cox (MATES in Construciton) and Chris Lockwood (MATES in Construction).

Participation procedure
Should you agree for your company to participate in the pilot program and associated research, workers at worksites that you nominate to be a part of the pilot will be asked to complete up to three surveys, at three time points. These surveys are designed to be brief. They include questions to assess knowledge and attitudes about suicide prevention, suicide ideation, self-harm, general help-seeking behaviours, and social support structures. These first survey is to be completed when you first sign on to the MATES in Manufacturing pilot, with follow up surveys at 6 and 12 months.

All surveys will be paper based, and they will distributed and collected at participating worksites by members of the MATES field officer team. Site visits for data collection purposes will be arranged with company management ahead of time, at a date and time that is most convenient to management.

Company consent and individual worker consent
Your company will be provided with a consent form prior to participating in this research project. In signing and returning the consent form to a member of the research team, you will be

acknowledging that you agree for worksites that your company has nominated to participate in the MATES in Manufacturing research evaluation, and that you give consent for research survey data collected to be used for the research purposes described in this statement.

At the company level, your company's engagement with the MATES in Manufacturing pilot program is contingent upon your agreement to participate in the research evaluation. This is because all new MATES programs involving new industries must be carefully evaluated by an independent research team. However, individual workers at a participating site have the right to refuse to participate in research data collection. This will not affect their engagement with the MATES in Manufacturing pilot program.

All workers at a participating site will be provided with a consent form prior to participating in this research project. In signing and returning the consent form to a member of the research team they will be acknowledging that they voluntarily agree to participate, and that they give consent for their data to be used for the research purposes described in the Participant Plain Language Statement.

What are the possible benefits?
By participating in this research, your company will be contributing to our understanding of suicide literacy, help-seeking, and help-offering behaviours among workers in the Australian manufacturing sector. This knowledge can be used to inform the future development and refinement of workplace suicide prevention programs specific to the manufacturing sector.

Please note that during a data collection visit to nominated sites, any individuals who show indications of acute distress on baseline surveys will be offered help by MATES staff (which they may choose to accept or not), and they can also nominate on your survey form if they would appreciate a follow-up call from a MATES staff member. This is available to any participant who is currently going through a tough time, and who would like support. In this way, the research process will also support individual workers to gain access to timely support.

What are the possible risks?
Thinking about suicide and self-harm may cause feelings of distress, for company workers and company leaders. If you do feel distress, you can contact the MATES 24/7 National Helpline (1300 642 111) or Lifeline (13 11 14). You will be provided with confidential support and connected to outside sources of counselling.

We recognise that participating in this research requires staff at nominated sites to gather in person, collectively. To help keep participation COVID safe, the research team will keep up-to-date with the Chief Health Officer's public health directions, and we will make any necessary adjustments to the project as circumstances change. We will only conduct surveys in locations at nominated worksites where all participants are able to observe physical distancing requirements. We will ensure hand sanitisers are available for use, and we will have disposable masks on hand, should any staff like one.

The participation of your company in this research project requires staff at your nominated sites to commit their time, and we recognise that this can pose an inconvenience to you, other members of company management, and your staff. To minimise this inconvenience, we have kept the survey short: it should only take workers 10 to 14 minutes to complete. Additionally, we have detailed in this form that data collection will take place at three six-month intervals (0, 6 and 12 months). Within this timeframe, we will work with company and site managers to schedule a visit at a date and time that works best for all relevant parties.

What will happen to information about me?

Company level information will be kept confidential, and so too will individual participant details. During the project, only the research team will have access to information about your company. Any data transferred between researchers across collaborating universities will only be done so using password-protected cloud storage and with all identifying information removed. Project data will be kept on a password-protected computer in a secure location at the MATES Australia office, and at Deakin University. We will keep this data for at least 5 years after the last publication arising from the project. This data is critical to ongoing evaluation of suicide prevention interventions, and efforts to save lives in the manufacturing industry rest on having good data. Therefore, the data may be used again by the research team for future evaluation efforts about suicide prevention among people working in manufacturing.

Does my company have to take part?

The MATES Australia National Board has passed a resolution that new pilot programs trialled in new industries must be subjected to an independent research evaluation. This is important for ensuring program fidelity, and for rigorously examining if the program adaptation in a new setting has been viable. For this reason, your company's participation in the MATES in Manufacturing pilot program is contingent upon your participation in this research evaluation.

In saying this, however, the participation of individual company staff members is completely voluntary. This means that when we visit a site for research data collection, individual workers can refuse to complete the survey if they do not wish to participate. This will not affect their participation in the MATES in Manufacturing pilot program. Additionally, a worker may withdraw from the research project at any time without penalty. Once data has been collected, it will be analysed and the results will be included in publications and presentations. However, if a worker chooses to withdraw from the project at any point, any unprocessed data that they have provided will not be included in publications and presentations.

The details of who does, and who does not, participate in the research evaluation will be kept confidential. Additionally, the data of workers who participate in the project will be kept confidentially, and securely.

Will I hear about the results of this project?

All participating companies will be provided with a report on completion of this project regarding the key project findings. Additionally, our team will provide your company with a plain English summary of outcomes, which can be shared with all staff.

We will also use data collected during this project to publish articles in academic journals, present at conferences, and to advocate for improvements in suicide prevention policy and practice.

Who is funding this project?
This project is funded by a *National Health and Medical Research Council Million Minds, Mission Suicide Prevention 2019* grant.

Where can I get further information?
If you have any questions, or would like more information, please contact responsible researcher, Professor Tony LaMontagne (03 9244 6802; tony.lamontagne@deakin.edu.au).

Who can I contact if I have any concerns about the project?
This research project has been approved by the Human Research Ethics Committee of Deakin University (ID #2021-xxx). If you have any complaints about any aspect of the project, the way it is being conducted or any questions about your rights as a research participant, then you may contact: The Human Research Ethics Office, Deakin University, 221 Burwood Highway, Burwood Victoria 3125, Telephone: 9251 7129, research-ethics@deakin.edu.au. Please quote project number [2021-***XXX]***. All complaints will be treated confidentially. In any correspondence, please provide the name of the research team or the name or ethics approval number of the research project.

Company Consent Form

MATES in Manufacturing: A Workplace Suicide Prevention Cluster RCT **Responsible Researcher:** Professor Anthony LaMontagne (Deakin University)

Additional Researchers: Dr Tania King (University of Melbourne), Neil Hall (Western Sydney University), Laura Cox (MATES in Construction) and Chris Lockwood (MATES in Construction)

1. I consent to participate in this project, the details of which have been explained to me, and I have been provided with a written plain language statement to keep.

2. I understand that the purpose of this research is about suicide literacy, help-seeking, and help-offering behaviours among people employed in the manufacturing sector.

3. I understand that my company's participation in the MATES in Manufacturing pilot program is contingent upon the company's participation in this research evaluation.

4. I understand that my company's participation in this project is for research purposes only.

5. I acknowledge that the possible effects of participating in this research project have been explained to my satisfaction.

6. In this project, company workers of participating worksites will be asked to complete an initial paper-based survey which will be distributed by a member of the research team at work sites that I have nominated, on behalf of the company, to participate in the MATES in Manufacturing pilot program. Workers will also be asked to complete follow-up surveys at 6 and 12 months.

7. I understand that the participation of individual workers is voluntary and that they are free to withdraw from this project at any time without explanation or prejudice. Workers can withdraw unprocessed data that they have provided.

8. I understand that the data from this research will be stored at Deakin University and will be retained for at least 5 years after the last publication is released from this project.

9. I have been informed that this research will be conducted in line with COVID safe requirements, and that COVID safe measures will be adjusted in line with the Chief Health Officer's public health directions, as these are issued.

10. I have been informed that the confidentiality of the information I provide -or the company workers provide - will be safeguarded subject to any legal requirements. All research data collected with my company will be password protected and accessible only by the named researchers. Any data transferred between researchers across different universities will only be done so using cloud storage and with all identifying information removed.

11. I understand that after I sign and return this consent form, it will be retained by the researcher.

**Name of Organisation:**	
**Name of Representative:**	
**Position of Representative:**	
**Signature of Representative:**	
**Mobile number:**	
**Date:**	

Withdrawal of Consent Form: Organisation

MATES in Manufacturing: A Workplace Suicide Prevention Cluster RCT

Responsible Researcher: Professor Anthony LaMontagne (Deakin University)

Additional Researchers: Dr Tania King (University of Melbourne), Neil Hall (Western Sydney University), Laura Cox (MATES in Construction) and Chris Lockwood (MATES in Construction)

Purpose of this Document

This document should be used if:

1. your organisation previously provided written consent by signing the *Organisational Consent Form (MATES in Manufacturing: A Workplace Suicide Prevention Cluster RCT)*. By signing that form, you provided consent for authorised researchers to conduct evaluation activities with your employees for research approved by a Human Research Ethics Committee, and
2. your organisation now wishes to withdraw that consent.

Withdrawal of Consent

On behalf of the organisation, I hereby withdraw consent for authorised researchers to conduct any further evaluation activities with employees, or to use previously collected employee survey data and contact details for research related to *MATES in Manufacturing: A Workplace Suicide Prevention Cluster RCT*. I understand that if any data has already been processed and included in reports or publications that it will not be possible to withdraw this data, however unprocessed data may still be withdrawn

On behalf of the organisation, I understand that our relationship with MATES in Construction, and universities of authorised researchers (Deakin University, University of Melbourne, Western Sydney University) will not be affected by our decision to withdraw organisational consent.

Contact Regarding Participation in Future Research

On behalf of the organisation, I hereby withdraw organisational consent to being contacted by a member of the authorised research team about participation in the *MATES in Manufacturing: A Workplace Suicide Prevention Cluster RCT.*

Name of Organisation: ____________________

Name of Representative: ____________________

Position of Representative: ____________________

Signature of Representative: ____________________

Date:
