# Supplementary material for: Evaluation of a workplace suicide prevention program in the Australian manufacturing industry: protocol for a cluster-randomised trial of MATES in manufacturing
Source: BMC Psychiatry. 2022 Dec 19;22:799. doi: 10.1186/s12888-022-04464-3 (PMC9761021; doi:10.1186/s12888-022-04464-3)
Supplement: Supplementary file 3 — Additional file 3. [file 12888_2022_4464_MOESM3_ESM.pdf]

Memorandum

To: Prof Tony LaMontagne
School of Health & Social Development

B

cc

From: Deakin University Human Research Ethics Committee (DUHREC)

Date: 03 September, 2021

Subject: 2021-276

MATES in Manufacturing: a workplace suicide prevention cluster RCT

Please quote this project number in all future communications

DUHREC considered the application for this project at its meeting held on 16/08/2021 and found it to comply with the National Statement on Ethical Conduct in Human Research 2007 (Updated 2018).

DUHREC has granted approval for Prof Tony LaMontagne , School of Health & Social Development, to undertake this project from 3/09/2021 to 3/09/2025.

The approval given by the Deakin University Human Research Ethics Committee is given only for the project and for the period as stated in the approval. It is your responsibility to contact the Human Research Ethics Unit immediately should any of the following occur:

- Serious or unexpected adverse effects on the participants
- Any proposed changes in the protocol, including extensions of time.
- Any events which might affect the continuing ethical acceptability of the project.
- The project is discontinued before the expected date of completion.
- Modifications are requested by other HRECs.
- Any complaints are received by the research team, an external HREC or, in the event of overseas research, an external complaints contact. In the case of overseas research, the local complaints contact should be aware that, where appropriate, they can directly contact DUHREC if they are unable to resolve a complaint or would like assistance in resolving a complaint.

In addition you will be required to report on the progress of your project at least once every year and at the conclusion of the project. Failure to report as required will result in suspension of your approval to proceed with the project.

DUHREC may need to audit this project as part of the requirements for monitoring set out in the National Statement on Ethical Conduct in Human Research 2007 (Updated 2018).

Please note: if you have indicated that your project will be conducted while COVID-19 restrictions are in place, approval has been granted in line with the current restrictions. It is the responsibility of the principal investigator to remain aware of any changes to the restrictions and in the event that such changes make the approved research non-compliant with the restrictions, to either seek approval for a further modification to the project, or postpone the research until the restrictions are lifted.

Human Research Ethics Unit
research-ethics@deakin.edu.au
Telephone: 03 9251 7123

WESTERN SYDNEY UNIVERSITY

HUMAN RESEARCH ETHICS COMMITTEE

30 September 2021

Doctor Neil Hall

School of Social Sciences

Dear Neil,

Project Title: "Mates in Manufacturing Suicide Prevention Project. Measuring the impact of on-site peer-to-peer mental health training to improve help-seeking and help-offering behaviour"

HREC Approval Number: H14506

Risk Rating: Moderate

I am pleased to advise the above research project meets the requirements of the National Statement on Ethical Conduct in Human Research 2007 (Updated 2018).

Ethical approval for this project has been granted by the Western Sydney University Human Research Ethics Committee. This HREC is constituted and operates in accordance with the National Statement on Ethical Conduct in Human Research 2007 (Updated 2018).

Approval of this project is valid from 30 September 2021 until 30 March 2023.

This protocol covers the following researchers:

Neil Hall, Tania King, David Henry, Shravan Guntuku, Anthony LaMontagne, Laura Cox

Summary of Conditions of Approval

1. A progress report will be due annually on the anniversary of the approval date.

2. A final report will be due at the expiration of the approval period.

3. Any amendments to the project must be approved by the Human Research Ethics Committee prior to being implemented. Amendments must be requested using the HREC Amendment Request Form.

4. Any serious or unexpected adverse events on participants must be reported to the Human Research Ethics Committee via the Human Ethics Officer as a matter of priority.

5. Any unforeseen events that might affect continued ethical acceptability of the project should also be reported to the Committee as a matter of priority.

6. Consent forms are to be retained within the archives of the School or Research Institute and made available to the Committee upon request.

7. Approval is only valid while you hold a position or are enrolled at Western Sydney University. You will need to transfer your project or seek fresh ethics approval from your new institution if you leave Western Sydney University.

8. Project specific conditions:

There are no specific conditions applicable.

Please quote the registration number and title as indicated above in the subject line on all future correspondence related to this project. All correspondence should be sent to humanethics@westernsydney.edu.au as this email address is closely monitored.

Yours sincerely

G. Weidemann

Associate Professor Gabrielle Weidemann

Presiding Member,

Western Sydney University Human Research Ethics Committee
